# Successful Amputation at the Hip-Joint for Sarcoma of the Thigh

**Published:** 1880-07

**Authors:** S. D. Cross

**Affiliations:** Professor of Surgery in the Jefferson Medical College


### SUCCESSFUL AMPUTATION AT THE HIP-JOINT FOR SARCOMA OF THE THIGH.

By S. D. CROSS, M.D.,
Professor of Surgery in the Jefferson Medical College.

This case was that of a clergymau, 47 years of age, a resident of Texas, who consulted me on the 8th of September last, on account of a diseased condition of the right thigh. He first noticed in 1867 a little tumor about the size of a nutmeg on the inner part of the limb. The tumor gradually increased in size, and when removed in 1871 it weighed one pound and a quarter and was pronounced by the operator to be of a fatty nature. The wound soon healed, and the gentleman returned to his accustomed occupation as a preacher. A new growth, however, before long appeared in the line of the cicatrix, and in the latter part of the same year another operation became necessary. Nothing further was done in the way of surgical interference until April, 1878, when another growth of considerable size was removed, intermixed with a large quantity of clotted blood, and having, like the preceding ones, the appearance of a mass of fat. A fourth operation was performed in the latter part of November of the same year. During the present year four more excisions have been performed,—one in the early part of February, the second on the 29th of March, the third on the 18th of June, on the last on the 5th of August. It will thus be perceived that there were altogether eight operations performed. During the last twelve months the recurrence of disease was rapid, although the wound always healed very kindly. Much coagulated blood was present in the more recent growths, and, what is remarkable, the more solid portions of them had always the appearance of ordinary fatty tumors. Another singular feature of these growths was the entire absence of pain until a very recent period. Of late there was a good deal of aching in the limb, especially after traveling in a railway car, but the local suffering was never severe. When the patient arrived in this city the thigh was much enlarged and ulcerated at at several points; there was a good deal of thin, sanguinolent discharge, and the limb was so stiff as to render it impossible to extend it.

The general health was good, but there was with a small and frequent pulse great emaciation, and anodynes were required to procure sleep and rest. The appetite was considerably impaired. As there was no constitutional involvement, so far as could be determined by a careful exploration of the chest and abdomen, nor any enlargement of the lymphatic glands of the groin, it was deemed best to afford the patient a chance for his life by removal of the limb at the hip-joint. After a thorough rest of twelve days, during which the patient was fed upon milk-punch, beef-tea, and other appropriate food, the operation was accordingly performed on the 20th of September, in the presence of Professor Acland, of Oxford, England, and of the pupils of the Jefferson Medical College, Drs. Brinton, Levis, and S. W. Gross kindly assisting.

The circulation in the limb, enveloped in Esmarch's bandage, so as to press out the blood, was controlled by the abdominal touriquet originally employed in this operation by Professor Joseph Pancoast in 1860 in a case of amputation at the hip-joint at the Pennsylvania Hospital. The operation as performed by me on the 20th ultimo, was, as some of you who were present on the occasion are aware, by cutaneous flaps taken from the inner and outer sides of the limb, the latter being by far the larger, as the disease had encroached less in that direction than in the other. As little muscular substance was left as possible. Only three arteries, in addition to the femoral, required to be ligated. After waiting fully one hour and a quarter, when all oozing of blood had ceased, the wound was loosely closed with four ordinary sutures and a long needle, the latter being inserted in such a manner as to approximate the deep portions of the flaps. A few narrow strips of adhesive plas- were applied to the upper part of the wound, the lower angle of which was left largely open to facilitate drainage. With a similar object in view, the most dependent portion of the outer flap was pierced by a free incision, and the opening thus made traversed by a piece of tape. Two hours after the patient had been placed in bed, a slight hemorrhage occurred from a muscular branch of the upper and inner portion of the wound, requiring the removal of a few sutures and the long pin, the latter of which was not again inserted.

Hardly an ounce of blood was lost in the operation, which was well borne by the patient. The bladder had been well emptied previously, and forty drops of laudanum had been administered an hour before the operation. The patient vomited once from the effects of the ether shortly after the operation was over. As soon as the stomach was able to bear it, milk-punch was given in small quantities, repeated every ten minutes, and kept up during the night along with beef essence. Black drop was also given to promote sleep, which soon became sound and refreshing. With the exception of the slight bleeding previously referred to, no untoward symptoms occurred after the operation. For the first thirty-six hours there was a free discharge of serosity, followed at the end of this time by tolerably free suppuration, which has continued up to the present time, sixteen days since the amputation. The patient has suffered no pain in the stump, and only a few times was there any spasm. He has taken ten grains of quinine daily since the operation. The bowels move daily of their own accord, and his spirits have all along been excellent, encouraged as he has been by the conviction that he is going to recover. One of the main objects during the treatment has been the administration at short intervals of nutritious food and drink. Beef-tea and milk-punch are the articles that have been mainly relied upon, assisted since his appetite has returned by beefsteak. The quantity of whisky daily consumed has been, until within the last few days, an average of about twelve ounces.

*October* 14.—This morning, twenty-four days after the operation, the ligature on the femoral artery was found lying loose in the wound, no attempt having been made at any time to remove it lest hemorrhage should be provoked.

*November* 3.—The wound is now nearly healed. The man's appetite is good; he sleeps well, and is in excellent spirits. The pulse, however, is still too frequent, and the system refuses to accumulate fat.

After the removal of the limb a careful dissection of the thigh disclosed the existence of three separate and distinct tumors, each nearly of the volume of an ordinary fist, of a whitish appearance, and a firm, dense consistence. The sectional surface exhibited a number of bloody points indicative of the presence of vessels. The tumors were non-encapsuled, and had evidently had their origin in the intermuscular connective tissue, as the muscles and thigh-bone were perfectly sound. A microscopic examination showed that the morbid growths are spindled-celled sarcomata, or what Sir James Paget has described under the name of recurrent fibromas.

*December* 28.—The patient late in November was sent to the sea-side at Atlantic City for change of air and scene. He returned at the end of eight days much improved in every respect, and then spent three weeks very advantageously at Allentown. He is now in Maryland in the enjoyment of good health and spirits, and expects in a short time to return to his home in Texas. The stump is well shaped and free from pain, but there is still in the direction of the acetabulum a slight discharge of thick yellow matter.

*June* 24, 1880.—I saw my patient a fortnight ago, plump, fat, and in excellent health, having gained upwards of twenty pounds in weight. He has for several months past preached every Sunday, and on several occosians twice on the same day, without any unusual fatigue. The stump is well shaped and thoroughly cicatrized, except at a little point over the acetabulum, where there is still, at times, a very slight oozing. There is no appearance whatever of a recurrence of disease.

